# Challenges in Clinical Decision-Making for Nurses in Neonatal Intensive Care Unit (NICU): A Qualitative Study

**DOI:** 10.1155/ccrp/6686680

**Published:** 2025-11-14

**Authors:** Fateme Mohammadi, Maryam Bahmanyar, Parisa Sabetsarvestani, Mostafa Bijani

**Affiliations:** ^1^Department of Nursing, Chronic Diseases (Home Care) Research Center and Autism Spectrum Disorders Research Center, Hamadan University of Medical Sciences, Hamadan, Iran; ^2^Department of Pediatrics, Fasa University of Medical Sciences, Fasa, Iran; ^3^Department of Medical Surgical Nursing, School of Nursing, Fasa University of Medical Sciences, Fasa, Iran

**Keywords:** clinical decision-making, critical care, neonatal, nurses, qualitative study

## Abstract

**Objective:**

Clinical decision-making is one of the most difficult responsibilities of healthcare personnel in the neonatal intensive care unit (NICU). Meanwhile, nurses in NICU are facing countless challenges to take care of these neonates due to complex clinical conditions, prematurity, low birth weight, and physiological instability of the neonates, making clinical decision-making difficult. Thus, it is essential to identify and investigate the challenges in clinical decision-making for nurses in NICU. The aim of the present study was to explore the challenges that nurses face in clinical decision-making in NICU in southern Iran.

**Methods:**

The present study is a qualitative study with the content analysis in Iran from February 2023 to January 2024. Twenty-one NICU nurses participated in this study. Data were collected from individual, in-depth, and semistructured interviews. The interview questions, according to the opinion of the research team, were designed around the research question: “What are the challenges in clinical decision-making for nurses in NICU?” In order to analyze the data, the researchers used the conventional content analysis method.

**Results:**

The means of the participants' ages and work experience were 36.52 ± 5.71 and 12.45 ± 5.83 years, respectively. Three main themes with nine subthemes were obtained in this study. The main themes were “inadequate clinical competence” (lack of knowledge about caring for neonates, lack of clinical skill and experience, and shortage of nurse practitioners in NICUs); “poor self-efficacy” (poor self-confidence, inefficient stress management, and lack of motivation); and “resistance to change” (physician-centeredness, lack of organizational support, and ambiguities about legal rights).

**Conclusion:**

The findings of the study revealed that lack of knowledge, poor clinical skills, and not having a master's degree in neonatal intensive care adversely affect nurses' self-confidence in providing care and making clinical decisions. Also, the common belief that physicians are superior to nurses in the organizational culture and lack of support for nurses undermines nurses' motivation for enhancing their competence and participating in clinical decision-making. Based on the findings, managers and policy makers, by providing a supportive environment, as well as improving the knowledge and clinical skills of nurses, can encourage them to participate in clinical decision-making.

## 1. Introduction

The neonatal intensive care unit (NICU) uses advanced equipment and skilled staff to effectively offer specialized care for neonates who require immediate medical attention [1]. Clinical decision-making refers to the interventions and strategies care providers take to change patients' current clinical conditions to the ultimate desired conditions. It consists of three basic stages: raising, understanding, and confirming the problem; suggesting and evaluating alternative solutions; and selecting and implementing the best solution [2]. As with the nursing process, the process of clinical decision-making starts with collecting data about the problem and ends with evaluation [3]. Clinical decision-making in nursing represents a goal-directed, analytic process that is central to professional practice. It is through this process that nurses, drawing on theoretical knowledge and accumulated clinical experience, identify and define patients' problems, arrange these problems in order of priority, and select the most appropriate therapeutic interventions and management strategies [4]. NICU nurses face the burden of achieving expertise in a range of complicated tasks spanning several domains in order to respond to the rapidly changing health conditions of newborns, and they are required to have excellent clinical decision-making skills to provide timely, accurate nursing care depending on the newborns' conditions [5]. However, the dynamic and changing conditions in nurses' work environment, combined with the uncertain and unstable conditions of patients, especially in acute care settings such as NICU, demand that nurses should be competent decision-makers and be able to blend their technical skills and professional knowledge to make proper clinical judgments about their patients' health status [6].

In the NICU, nurses care for premature and critically ill neonates, interact with parents, and make clinical decisions regarding the treatment of neonates in life-threatening conditions [7]. The nurses in special care units, especially NICU, are faced with many challenges in making clinical decisions [7, 8]: complications in the neonates' clinical status, prematurity, low weight, heart diseases, respiratory distress syndrome, asphyxia, meconium aspiration syndrome, sepsis and infection, convulsions, hypoglycemia, and extensive changes in their hemodynamic conditions require nurses to make quick decisions and take interventions as resuscitation, intubation, hypothermia therapy (neonatal cooling), and total parenteral nutrition (TPN) therapy within the shortest possible time [7, 8].

Neonates, especially premature neonates, are among the most vulnerable groups whose survival depends on effective nursing interventions [9]. According to the Ethiopian Mini-Demographic Health Survey (Mini-EDHS) study, newborn mortality is likely to increase, rising from 29 per 1000 live births in 2016 to 33 per 1000 live births in 2019 [10]. In Iran, exact statistics are not available, but studies show that 12%–15% of neonates are admitted to the NICU every year, with most of them constituting about 10% of neonates who were born prematurely [11].

Thus, the healthcare team aims to not only keep premature neonates alive but also to keep their quality of survival high [12]. Providing care in NICUs can be very stressful for the healthcare team [13] because managing premature neonates with special and complicated needs is a very difficult task and poses many challenges in care [14]. One of the greatest challenges for NICU nurses is overcoming the obstacles to making the best clinical decision [15]. According to Namnabati et al., lack of experienced and well-trained nurses, lack of specialized equipment, conflicts between nurses and physicians, as well as fatigue and burnout are among the major challenges which adversely affect nurses' clinical decisions in NICUs [7]. Dagla et al. reported that health professionals' understanding and skill in dealing with moral conflicts in NICU can contribute to clinical decision-making, NICU policies, and support strategies, eventually enhancing the quality of intensive care provided to neonates [15].

A study by Tubbs-Cooley indicated that the pressure and workload of NICU nurses is significantly related to their clinical decisions and subsequently the number of missed nursing care. Specifically, the more serious and complex the clinical condition of the infants admitted to the NICU, the greater the pressure and workload these nurses are faced with. Subsequently, they face many challenges in choosing the mode of mechanical ventilation, decision-making about caring in end-of-life, continuing drug therapy and newborn discharge, all heavily affecting their clinical decision-making [16].

Other important challenges in clinical decision-making in NICU are family-centered care and family support, issues that are often neglected and no systematic programs have been developed to address them. The challenges in clinical decision-making in NICU are influenced by organizational atmosphere and culture, as well as the level of legal authority and duties of nurses. [14–16]. Meanwhile, qualitative studies help explain and describe the phenomena, the factors affecting them and the challenges in the cultural context and from the perspective of the people who are involved with that phenomenon. Thus, conducting a qualitative study can be effective to explain the clinical decision-making challenges of nurses in NICU departments [17].

## 2. Background in Iran

NICU nurses in Iran hold a Bachelor of Science in Nursing (BSN), a Master of Science in Nursing (MSN), or a doctoral degree in nursing (PhD). Prior to their assignment to the NICU, nurses are expected to possess clinical experience in neonatal critical care and to have completed relevant training courses. In Iran, NICUs are classified into three levels (Level I: Basic Newborn Care, Level II: Advanced Newborn Care, and Level III: Subspecialty Newborn Care). Nursing care in the NICU is delivered under the supervision of neonatology specialists, and nurses do not practice autonomously; advanced practice roles such as nurse practitioners (NP) are not established in Iran. For professional development, annual in-service training courses are provided, and participation is mandatory.

Accordingly, in view of the important role of NICU nurses' clinical decision-making skills in preventing complications in neonates' clinical conditions, which lead to extra expenses for the healthcare system and the families, as well the impact of hospital environment and culture on nurses' clinical decisions and lack of qualitative research in this area, the aim of present study was to investigate the challenges in clinical decision-making for nurses in NICU in Iran.

## 3. Methods

The present study was a qualitative research conducted via the content analysis, from February 2023 to January 2024. Content analysis was also used when little information was available about the concept [17].

The researchers used this approach since the challenges of ethical judgment of interviewers in NICUs in Iran have not yet been fully explained and described, and to answer the research question, “What are the challenges in clinical decision-making for nurses in NICUs?” The reporting of the study was based on the consolidated criteria for reporting qualitative research (COREQ) checklist [18].

### 3.1. Participants

The study population consisted of nurses who worked in NICU. In this study, 31 level II and level III nurses from NICU were invited to participate in the study. The inclusion criteria were: having at least 2 years of experience of practice in NICU, being Iranian, as well as speaking and understanding Persian. The participants were selected through purposeful sampling method and were invited to participate in this study. The corresponding author went to the nursing office in the hospitals and showed ethics approval for this study.

The head of the nursing office was asked to allow placing the notice of participation in the study on the board of nurses in the hospitals. Also, if they wished, nurses should visit the nursing office and register their contact number. Next, 31 nurses announced their readiness and allowed their mobile numbers to be given to the corresponding author. Eight participants did not meet the criteria for entering the study (6 nurses had less than 2 years of work experience, 2 nurses did not want to participate in the study). Totally, 23 nurses participated in this study. The participants were selected via purposeful sampling, a method commonly used in qualitative research to identify cases with rich information about the phenomenon under study. Information-rich cases are individuals who can provide important information about the research subject. By studying information-rich cases, researchers can gain an in-depth understanding, rather than empirical generalizations [17]. While conducting interviews, two nurses refused to continue the interview. Thus, 21 nurses, who met the inclusion criteria of the study, were interviewed. The interviews continued until reaching saturation. Saturation happens when no new categories could be extracted with the categories being saturated in terms of characteristics and dimensions [19].

### 3.2. Data Collection

Data were collected with semistructured interviews and field note. The time and place of the interview was decided based on the opinion of the participants. All interviews were conducted face-to-face at times which were convenient for the participants in a unit's conference room with due regard for participants' privacy. Only the researcher and the participant were present during each interview, and no other staff members were admitted, enabling participants to respond to questions candidly. Interviewees were assured that their information would be treated confidentially and that the interview data would be used solely for the purposes of this research. At the beginning of each interview, the purpose of conducting the research was explained to the participants. The participants were assured about their anonymity and confidentiality of their information, and written informed consent was taken from them to participate in the study. The interview questions were “what is the meaning of clinical decision-making in your opinion?”, “what factors influence clinical decision-making?”, “what situations and environmental conditions affect your clinical decisions?”, and “what are the challenges in clinical decision-making for nurses in NICUs?”. Next, based on the respondents' answers, follow-up questions were asked to increase the clarity of the information; these questions included, “Can you explain further?”, “What do you mean by that?”, and “Can you give an example?”. Twenty-one semistructured interviews were done. Each interview lasted 40 to 50 min. The interviews were recorded with the oral and written consent of the participants. Immediately after completion, each interview was listened to several times and then transcribed by the corresponding author who tried to gain a general understanding of the subject of the study. Next, the collected data were analyzed by all researchers and the next interview was designed accordingly. Interviewing continued until on new subthemes could be extracted and the data were considered saturated. The data were analyzed by the MAXQDA software [20].

### 3.3. Data Analysis

Data were analyzed using Graneheim and Lundman's method in 5 steps as follows: (1) The recorded speeches were transcribed; (2) By carefully studying the manuscripts, the researcher tried to find the external and internal elements of the speech. In other words, immersion in the data was done to first gain the participants' understanding; (3) By re-reading the scripts, the semantic units were identified as sentences or paragraphs from the statements and texts of the interview; (4) Then, the initial codes or open codes were extracted from it. In open coding, the text of each interview was read several times and the main sentences were extracted and recorded as codes. The related codes extracted based on similarity were then placed in subcategories; and (5) Subcategories were merged to create the final themes [21].

### 3.4. Rigor

To enhance the credibility and accuracy of the data, the researchers used a combination of methods: semistructured interviews, prolonged engagement with the data, member checking, and peer checking. For member checking and peer checking, three of the participants and four peers were presented with the extracted codes, subthemes, and themes, and asked to verify if the findings were consistent with their perceptions. The methods of coding, extraction of subthemes, and classification of the subthemes into themes were verified by three of the participants and four experts. Furthermore, in order to minimize bias in collecting, analyzing, and coding of the data, thereby boosting the validity of the findings, the researchers limited the literature review at the beginning of the study. As for confirmability, via exact recording of the participants' narratives and detailed reporting of the study results, the researchers have made follow-up for other researchers possible [20].

### 3.5. Ethical Considerations

All participants gave written informed consent to participate in the study. The participants were assured about their anonymity and confidentiality of their information. The present study was conducted according to the principles of the revised declaration of Helsinki, a statement of ethical principles which guide researchers in medical research involving human subjects. Further, the study was approved by the Institutional Research Ethics Committee of Fasa University of Medical Sciences, Fasa, Iran (Ethical code: IR.FUMS.REC.1401.156).

## 4. Results

In the present study, 21 nurses were interviewed. The means of the participants' ages and work experience in NICU were 33.61 ± 5.71 and 12.45 ± 5.83 years, respectively. The other demographic features of the participants are reported in Table 1. In this study, 1013 codes, which were 23 subcategories, 9 categories, and 3 main themes, as follows: “in adequate clinical competence,” “poor self-efficacy,” and “resistance to change,” were extracted from the data. The themes and categories were extracted based on content analysis (Table 2).

### 4.1. Inadequate Clinical Competence

The interviewed nurses stated that they had not acquired effective clinical skills and competence for practice in NICUs during their education and training. Many of the nurses had never been in an NICU, nor had they provided care to neonates in the course of their undergraduate programs. They also mentioned that only a few of the subjects in their curriculum were about caring for neonates and interacting with their families. Thus, they had not acquired adequate clinical competence for working in NICUs. Lack of effective clinical competence had an adverse effect on the nurses' clinical reasoning and, by extension, the quality of care. The theme of inadequate clinical competence consists of three categories: lack of knowledge about caring for neonates, lack of clinical skill and experience, and shortage of nurse practitioners in NICUs.

#### 4.1.1. Lack of Knowledge About Caring for Neonates

The nurses who were interviewed in the present study mentioned that their knowledge of neonates' stages of development and diseases as well as methods of caring for neonates and interacting with their families was limited. The nurses' bachelor's degree program offered only five to six credits on neonates and did not prepare them for providing care to neonates. Therefore, the nurses did not possess adequate clinical reasoning skills in caring for neonates.

*“When I was an undergraduate, there were about 5 or 6 course credits for pediatric diseases and only one credit for neonatal disorders. Well, naturally, what we know about neonates' diseases and how we should care for them or interact with their parents is limited. I simply don't have enough knowledge. And without enough relevant knowledge, you can't have a good understanding of your patients' conditions and provide the best care”* (participant 5).

#### 4.1.2. Lack of Clinical Skill and Experience

The participants stated that in the course of their undergraduate studies, they had never been in NICUs or provided care to newborn and premature neonates. Practicing in NICUs and giving care to premature as well as undersized neonates which are attached to a variety of devices require great clinical skills and competence. However, most nurses acquire these skills in the years when they are working, which inevitably affects their clinical reasoning.

*““Due to the limitation of my training fields during my undergraduate course, I did not enter NICU department. I'd never seen a baby hooked up to equipment several times its own size, let alone give care to a newborn in NICU. … Well, caring for this group of patients needs a great deal of clinical skills and experience. Without the right skills and knowledge, you can't make the best decision about what kind of care to give to these neonates based on their conditions”* (participant 8).

#### 4.1.3. Shortage of Nurse Practitioners in NICUs

The nurses who provided care to neonates in NICU mentioned that the number of nurses with a master's degree in neonatal intensive care (nurse practitioners) in their units was very small. Moreover, there were not any nurses with a master's degree in NICU (nurse practitioners) in many of their work shifts. Meanwhile, it is essential that a nurse practitioner should be present in every shift. This is because registered nurses can observe the care and make clinical decisions of the nurse practitioner, which is of utmost importance in training for registered nurses. Also, nurse practitioners help other registered nurses in providing care and making clinical decisions for hospitalized neonates, which develops the skills and self-confidence of registered nurses.

*“There are just a few nurse practitioners in our unit and in many of the shifts, they are absent. But when I* am *with a nurse practitioner in my shift, I have peace, and by watching them, I learn how I should give care and make clinical decisions in different situations. … Also, I can ask them my questions. And when a patient's conditions become critical, they quickly help me and tell me what to do, which improves my skills and self-confidence”* (participant 14).

### 4.2. Poor Self-Efficacy

The nurses in the present study stated that they did not have enough self-confidence to care for premature neonates in NICUs and were not able to properly manage stressful situations. Meanwhile, work overload and poor prospects of promotion in their jobs made them reluctant to improve their professional competence. These factors resulted in the nurses' poor self-efficacy, which in turn adversely affected their clinical reasoning and decision-making. The theme of poor self-efficacy consists of three categories: poor self-confidence, inefficient stress management, and lack of motivation.

#### 4.2.1. Poor Self-Confidence

The participants stated that they did not have enough self-confidence to provide care to premature neonates and make effective clinical decisions.

*“I've been working in this unit for seven years. … Caring for neonates, especially the ones who are sick or premature, is really stressful. Sometimes, these neonates have come after years of infertility and are the only hope of their families. I honestly don't have the self-confidence to tell myself that this is the best decision a nurse can make for this baby in this situation and act accordingly. My poor self-confidence makes me doubt my clinical decisions”* (participant 10).

#### 4.2.2. Inefficient Stress Management

The NICU nurses who were interviewed in the present study mentioned that the work conditions in NICUs were very stressful and that caring for undersized neonates attached to a variety of medical equipment is a demanding task. Thus, work stress management is a crucial factor in reducing stress in the workplace and making right clinical decisions.

*“The work environment here is very stressful. Premature neonates are really small and delicate. Performing any kind of intervention, especially invasive interventions, can induce great stress to nurses. … When you feel stressed, you can't judge situations well and make the right decision”* (participant 16).

#### 4.2.3. Lack of Motivation

Lack of motivation is one of the most significant categories extracted from the data. The interviewed nurses stated that years of hard work as well as effort to improve their knowledge and skill had not resulted in a change in their work conditions, salary, or even social and organizational status. They lacked motivation for becoming involved in making challenging clinical decisions and preferred to follow the orders of their superiors.

*“I've worked hard in this unit for many years … and expanded my knowledge and skills. … I remember once I made a clinical decision for a newborn in my charge. But my superior ignored my decision with contempt. When the patients' conditions were discussed in a meeting, my decision was confirmed. … I wondered ‘I used my years of experience to make this decision and gave logical reasons to explain it, but they barely accept it.' I feel responsible for my patients. But the way we are treated by our superiors make us lose motivation for engaging in decision-making, so we just follow orders”* (participant 20).

### 4.3. Resistance to Change

The participants stated that the dominant organizational culture was characterized by a physician-centered attitude, lack of support for nurses, and ambiguities about the nurses' legal rights and liability for their clinical decisions, all of which challenged NICU nurses in making clinical decisions. Resistance to change consists of three categories: physician-centeredness, lack of organizational support, and ambiguities about legal rights.

#### 4.3.1. Physician-Centeredness

The participating nurses mentioned that a physician-centered attitude prevailed in their organization, and they were sometimes not allowed to participate in clinical decision-making, whereby their opinions and clinical experience as well as knowledge were ignored. However, since they are the ones who execute the orders given by physicians, they are liable for any negative consequences of those clinical decisions.

*“I'm a nurse practitioner with 22 years of work experience …. On the clinical rounds, nobody pays attention to my comments and ideas. When I say that a certain intervention might be more effective, nobody listens. I must just follow the* physicians' *written orders. When the baby's condition deteriorates, then the intervention I'd suggested earlier becomes an order. At times like this, I feel sad and guilty. … This physician-centered attitude is the biggest obstacle to nurses' participation in clinical decision-making”* (participant 1).

#### 4.3.2. Lack of Organizational Support

The interviewed nurses mentioned that the organization did not support them sufficiently, especially in the case of making clinical decisions and the consequences of those decisions. Therefore, the nurses were not willing to participate in the process of making clinical decisions. They stated that when nurses know that the organization and organizational rules support them in making clinical decisions in front of patients, physicians, and other medical staff, they are more motivated to participate in clinical decisions.

“The organizational atmosphere does not support nurses or their decisions and actions. Typically, hashtags that defend patients in front of doctors, supervisors, etc. Usually, when nurses defend patients in front of doctors, supervisors, etc. and talk about treatment and care mistakes, nurses and their clinical decisions are not supported at all. The nurses know there is no one to support them, so they prefer not to take part in clinical decision-making” (participant 7).

#### 4.3.3. Ambiguities About Legal Rights

Another category of the present study is ambiguities about nurses' duties and legal rights. The nurses stated that they are required to directly execute medical-clinical interventions without having a role in clinical decision-making. On the other hand, there are ambiguities about their legal rights in the organization's job descriptions, making the nurses confused about performing their duties, participating in care, and making clinical decisions.

*“I don't exactly know what my powers and rights are in making clinical decisions. According to the organizational chart, my job is taking part in clinical rounds and making nursing decisions, but in reality, I have no role in making clinical decisions”* (participant 3).

## 5. Discussion

An investigation of the challenges in clinical decision-making for nurses in NICUs revealed that the obstacles these nurses are faced with fall into three themes: inadequate clinical competence, poor self-efficacy, and resistance to change.

Clinical competence is one of the important themes of the present study. Lack of clinical knowledge, skills, and experience as well as specialized training in caring for neonates in NICUs is the main cause of nurses' inadequate clinical competence and, by extension, incompetence in making clinical decisions. Similarly, Dombrecht et al. attributed lack of confidence in nurses' clinical diagnoses to their lack of knowledge, experience, and specialized training, which is a major obstacle to nurses' clinical decision-making in NICUs [22]. Said Hendy et al. reported that NICU nurses' knowledge of caring for premature neonates is not satisfactory, with an adverse effect on their decision-making competence and clinical performance [23]. Esmaelzadeh et al. attributed NICU nurses' poor decision-making competence and clinical performance to the lack of courses on providing care to neonates in the nursing curriculum and not giving undergraduate nursing students the chance to receive training in NICUs [24]. According to Lee et al., experienced nurses with specialized knowledge of caring for neonates have a better understanding of clinical situations and can, therefore, make better clinical decisions. These findings confirm that lack of clinical competence due to inadequate knowledge, skills, and specialized training is one of the primary causes of NICU nurses' failure to participate in making nursing clinical decisions [25].

Another theme extracted from the data in the present study has been poor self-efficacy. The nurses listed lack of self-confidence, inability to manage work stress, and lack of motivation due to their managers' disregard for their efforts as the main reasons for their sense of low self-efficacy. According to Anggraini et al., there is a significant positive correlation between nurses' self-efficacy and clinical decision-making skills in resuscitating neonates in NICUs. Since nurses are among the most important members of the treatment team, it is essential that their clinical decision-making skills be enhanced [26]. As for work stress, Mathebula et al. found that work stress in NICUs is one of the main factors which adversely affects nurses' self-efficacy and, hence, their ability to make clinical decisions [27]. A study by Choi and Kim showed that pediatric nurses' professional competence and self-efficacy have a significant impact on their clinical decision-making skills [28].

The third theme extracted from the data in the present study has been resistance to change. Organizational culture and climate, the organization's attitude to nurses and physicians, the extent of support for nurses, as well as clarity about nurses' position and legal rights affect nurses' willingness to participate in clinical decision-making [21, 26]. Other studies state that the scope of duties and legal rights of nurses are not clear, as well as the prevalence of negative attitudes toward their presence in making clinical decisions, which has caused nurses to be unmotivated to participate in clinical decision-making, which is caused by the lack of dynamism in the organizational culture, making nurses reluctant to make clinical decisions [26, 28]. Similarly, according to Feldman and Rohan, a dynamic organizational culture is the most important factor in encouraging NICU nurses to participate in clinical decision-making [29]. Meanwhile, Esmaelzadeh et al. mentioned poor interaction between physicians and nurses as a major obstacle to nurses' participation in clinical decision-making [24]. Arreciado Marañón et al. reported that one of the main reasons for nurses' limited participation in clinical decision-making is the physicians' and organization's inadequate support for them [30]. According to Rubic et al., making clinical decisions for neonates, especially severely ill neonates, is a very demanding task and it is essential that medical-healthcare organizations support caregivers and nurses in clinical decision-making [31]. Similarly, Rafiee et al. maintained that legal support for physicians and nurses can reassure them and make them willing to participate in clinical decision-making in NICUs [32]. In summary, lack of self-confidence, inadequate knowledge and skills in caring for neonates, a physician-centered organizational culture, and lack of support for nurses are the main challenges in NICU nurses' participation in clinical decision-making.

## 6. Limitations

One of the limitations of the present study is that data were collected exclusively via personal interviews. Other methods of data collection, including focus group and observation, could have enriched the findings of this work of qualitative research. Thus, it is suggested that future studies rely on more varied methods of data collection. The nurses who participated in the present study were selected from the NICUs of state hospitals. Therefore, it is suggested that future studies explore the challenges in clinical decision-making from the viewpoint of nurses in private hospitals.

## 7. Conclusion

NICUs are among the most stressful environments in hospitals. The nurses who are in practice in these units are faced with challenges in clinical decision-making which adversely affect their professional performance. Lack of knowledge, skills, and specialized training in caring for neonates seriously undermine nurses' self-confidence in providing care and making clinical decisions in NICUs. Meanwhile, the prevalence of a physician-centered attitude in the organizational culture and lack of support for nurses are among the major obstacles to nurses' participation in clinical decision-making. Managers and policy makers can use the findings of the present study to create a supportive environment for nurses and improve their knowledge and skill, thus encouraging nurses to make clinical decisions. Also, according to the findings of this study, managers and educational supervisors in hospitals can develop the knowledge, skills, and confidence of nurses by holding workshops on how to care for neonates and make clinical decisions in nursing care.

## Figures and Tables

**Table 1:** Individual characteristics of the participants.

Participants	Age	Sex	Educational level	Work experience in NICU (years)
P1	26	Female	Bachelor's degree in nursing	3
P2	39	Female	Master's degree in nursing	12
P3	34	Female	Bachelor's degree in nursing	9
P4	29	Female	Bachelor's degree in nursing	5
P5	33	Female	Master's degree in nursing	8
P6	39	Female	Bachelor's degree in nursing	15
P7	37	Female	Bachelor's degree in nursing	13
98	42	Male	Master's degree in nursing	17
P9	38	Female	Bachelor's degree in nursing	11
P10	32	Female	Master's degree in nursing	9
P11	27	Female	Bachelor's degree in nursing	5
P12	38	Female	Master's degree in nursing	10
P13	26	Male	Bachelor's degree in nursing	3
P14	37	Female	Bachelor's degree in nursing	11
P15	43	Female	Master's degree in nursing	17
P16	40	Female	Master's degree in nursing	15
P17	44	Male	Bachelor's degree in nursing	17
P18	39	Female	Bachelor's degree in nursing	11
P19	37	Female	Master's degree in nursing	12
P20	42	Female	Bachelor's degree in nursing	17
P21	48	Male	Bachelor's degree in nursing	22

**Table 2:** Themes and subthemes extracted from content analysis.

Themes	Subthemes
Inadequate clinical competence	• Lack of knowledge about caring for neonates
	• Lack of clinical skill and experience
	• Shortage of nurse practitioners in NICUs

Poor self-efficacy	• Poor self-confidence
	• Inefficient stress management
	• Lack of motivation

Resistance to change	• Physician-centeredness
	• Lack of organizational support
	• Ambiguities about legal rights

## Data Availability

The data supporting this study are available upon reasonable request from the corresponding author.

## References

[1] Chow S., Chow R., Popovic M. (2015). A Selected Review of the Mortality Rates of Neonatal Intensive Care Units. *Frontiers in Public Health*.

[2] Howe E. G. (2020). Beyond Shared Decision Making. *Journal of Clinical Ethics*.

[3] Santoro J. D., Bennett M. (2018). Ethics of End of Life Decisions in Pediatrics: A Narrative Review of the Roles of Caregivers, Shared Decision-Making, and Patient Centered Values. *Behavioral Sciences*.

[4] Park E. S., Cho I. Y. (2018). Shared Decision-Making in the Paediatric Field: A Literature Review and Concept Analysis. *Scandinavian Journal of Caring Sciences*.

[5] Kouravand Z., Aein F., Ebadi A., Yadegarfar G. (2019). Nurses’ Perception of Clinical Decision Making in Hospitals of Shahrekord University of Medical Sciences in 2019. *Journal of Clinical Nursing and Midwifery*.

[6] Boland L., Graham I. D., Legare F. (2019). Barriers and Facilitators of Pediatric Shared Decision-Making: A Systematic Review. *Implementation Science*.

[7] Namnabati M., Farzi S., Ajoodaniyan N. (2016). Care Challenges of the Neonatal Intensive Care Unit. *IJNR*.

[8] Mitchell S., Spry J. L., Hill E. (2019). Parental Experiences of End of Life Care Decision-Making for Children With Life-Limiting Conditions in the Pediatric Intensive Care Unit: A Qualitative Interview Study. *BMJ Open*.

[9] Shahraki Moghaddam E., Manzari Z., Ghandehari Motlagh Z. (2017). The Evaluation of Nurses Clinical Decision Making in Intensive Care Unit at the Teaching Hospitals of Mashhad. *Journal of Sabzevar University of Medical Sciences*.

[10] EPHI Ethiopia Mini Demographic and Health Survey 2019: Key Indicators. Rockville, Maryland, USA: EPHI and ICF 2019. https://dhsprogram.com/pubs/pdf/FR363/FR363.pdf.

[11] Kheiry F., Mohammadbeigi A., Afrashteh S. (2019). Factors Associated With Length of Stay in a Neonatal Intensive Care Unit in Iran. *Sri Lanka Journal of Child Health*.

[12] Connor D., Lynch H., Boyle B. (2021). A Qualitative Study of Child Participation in Decision-Making: Exploring Rights-Based Approaches in Pediatric Occupational Therapy. *PLoS One*.

[13] Goldhagen J., Clarke A., Dixon P., Guerreiro A. I., Lansdown G., Vaghri Z. (2020). Thirtieth Anniversary of the UN Convention on the Rights of the Child: Advancing a Child Rights-Based Approach to Child Health and Well-Being. *BMJ Paediatrics Open*.

[14] Sasazuki M., Sakai Y., Kira R. (2019). Decision-Making Dilemmas of Pediatricians: A Qualitative Study in Japan. *BMJ Open*.

[15] Dagla M., Petousi V., Poulios A. (2021). Neonatal End-of-Life Decision Making: The Possible Behavior of Greek Physicians, Midwives, and Nurses in Clinical Scenarios. *International Journal of Environmental Research and Public Health*.

[16] Tubbs-Cooley H. L., Mara C. A., Carle A. C., Mark B. A., Pickler R. H. (2019). Association of Nurse Workload With Missed Nursing Care in the Neonatal Intensive Care Unit. *JAMA Pediatrics*.

[17] Mohammadi F., Javanmardifard S., Bijani M. (2024). Women Living With Infertility in Iran: A Qualitative Content Analysis of Perception of Dignity. *Women’s Health*.

[18] Tong A., Sainsbury P., Craig J. (2007). Consolidated Criteria for Reporting Qualitative Research (COREQ): A 32-Item Checklist for Interviews and Focus Groups. *International Journal for Quality in Health Care*.

[19] Saunders B., Sim J., Kingstone T. (2018). Saturation in Qualitative Research: Exploring Its Conceptualization and Operationalization. *Quality and Quantity*.

[20] Mohammadi F., Farjam M., Gholampour Y., Tehranineshat B., Oshvandi K., Bijani M. (2020). Health Professionals’ Perception of Psychological Safety in Patients With Coronavirus (COVID-19). *Risk Management and Healthcare Policy*.

[21] Graneheim U. H., Lundman B. (2004). Qualitative Content Analysis in Nursing Research: Concepts, Procedures and Measures to Achieve Trustworthiness. *Nurse Education Today*.

[22] Dombrecht L., Piette V., Deliens L. (2020). Barriers to and Facilitators of End-of-Life Decision Making by Neonatologists and Neonatal Nurses in Neonates: A Qualitative Study. *Journal of Pain and Symptom Management*.

[23] Said Hendy A., Saad Al-Sharkawi S., Abd Al-Moniem I. I., Ibrahim I. (2020). Nursing Competency for Caring of High-Risk Neonates at Neonatal Intensive Care Unit. *Egyptian Journal of Health Care*.

[24] Esmaelzadeh F., Nematollahi M., Mehdipour-Rabori R., Bagherian B. (2020). Educational Challenges of Postgraduate Neonatal Intensive Care Nursing Students: A Qualitative Study. *Journal of Education and Health Promotion*.

[25] Lee H. N., Park J. H., Cho H. (2023). Developmentally Supportive Care Among Neonatal Intensive Care Unit Nurses in South Korea: Knowledge, Perceived Importance, Perception, and Perceived Competence. *Advances in Neonatal Care*.

[26] Anggraini I. R., Putra K. R., Setyoadi S. (2021). Influence of Communication, Collaboration, and Decisionmaking Skills on the Efficacy of Nurses in Conducting Neonatal Resuscitation. *International Journal of Public Health Science*.

[27] Mathebula M., Bopape M., Mutshatshi T., Ramavhoya T. (2024). Challenges Faced by Midwives Who Care for Neonates in Neonatal Intensive Care Units of Public Hospitals in Limpopo Province, South Africa. *The Open Public Health Journal*.

[28] Choi M., Kim J. (2015). Relationships Between Clinical Decision-Making Patterns and Self-Efficacy and Nursing Professionalism in Korean Pediatric Nurses. *Journal of Pediatric Nursing*.

[29] Feldman K., Rohan A. J. (2022). Data-Driven Nurse Staffing in the Neonatal Intensive Care Unit. *MCN: The American Journal of Maternal/Child Nursing*.

[30] Arreciado Marañón A., Fernández-Cano M. I., Montero-Pons L. (2022). Understanding Factors That Influence the Decision to Be Vaccinated Against Influenza and Pertussis in Pregnancy: A Qualitative Study. *Journal of Clinical Nursing*.

[31] Rubic F., Curkovic M., Brajkovic L. (2022). End-of-Life Decision-Making in Pediatric and Neonatal Intensive Care Units in Croatia—A Focus Group Study Among Nurses and Physicians. *Medicina*.

[32] Rafiee Z., Rabiee M., Rafati S., Rejeh N., Borna H., Vaismoradi M. (2020). Physicians’ and Nurses’ Decision Making to Encounter Neonates With Poor Prognosis in the Neonatal Intensive Care Unit. *Clinical Ethics*.

